# The Role of Generative Artificial Intelligence in Shaping University Students’ Learning Behavior: A Mixed-Method Research Based on the COM-B Model

**DOI:** 10.3390/bs16040577

**Published:** 2026-04-11

**Authors:** Rui Ma, Mingfei Guo

**Affiliations:** 1 Faculty of Artificial Intelligence Education, Central China Normal University, Wuhan 430079, China; manman0614@mails.ccnu.edu.cn; 2 School of Marxism, Central China Normal University, Wuhan 430079, China

**Keywords:** generative artificial intelligence, university students, learning behaviors, AI literacy, the COM-B model, CB-SEM, fsQCA

## Abstract

While GenAI is transforming education, it remains unclear how it shapes students’ behavior, especially concerning AI literacy. The purpose of this study is to examine which factors positively affect students’ learning behavior and whether AI literacy moderates this effect, using the COM-B model. An online survey of 438 participants was analyzed using covariance-based structural equation modeling (CB-SEM) and fuzzy-set qualitative comparative analysis (fsQCA). The CB-SEM results indicate that independent learning ability, receptive ability, learning environment, AI support equipment, and both intrinsic and extrinsic motivations significantly shape student learning behavior. Notably, AI literacy moderates the relationship between GenAI and learning behavior. Furthermore, fsQCA reveals seven configurations of these factors that favorably impact learning behavior. Together, these findings provide theoretical and practical insights for universities, highlighting actionable ways universities can support students’ adoption of GenAI.

## 1. Introduction

In recent years, artificial intelligence (AI) technology has made great strides and is gradually permeating most areas of human society. Among the many branches of AI, generative artificial intelligence (GenAI) shows outstanding research and application value. Unlike traditional AI, which relies on rule matching and pattern recognition, GenAI uses deep neural networks. It learns from massive data to capture patterns and generate new content (Ferrara, 2024; Mittal et al., 2024). The emergence of GenAI has expanded AI’s application boundaries and opened new possibilities for education. In higher education, GenAI improves students’ learning efficiency and creates opportunities for creativity and critical thinking (Tillmanns et al., 2025). For example, GenAI is transforming teaching by reallocating resources, fostering pedagogical innovation, and redefining educators’ roles (Sohail et al., 2025; Vassileva & Daneva, 2025; Zhai, 2024). These changes have inevitably impacted students’ learning process and behavior. Therefore, this study aims to investigate the impact of GenAI on students’ learning behavior.

When integrated into the curriculum, GenAI can enhance students’ learning experiences in many ways. For example, GenAI personalizes the learning process and adapts instruction to student needs and preferences by leveraging large datasets and machine learning algorithms (Leon, 2024; Anderson et al., 2025). Furthermore, by analyzing students’ weaknesses and learning patterns, it provides tailored feedback and suggests personalized study materials or exercises (W. Hu & Shao, 2025). In addition, GenAI assists teachers in assessing students’ progress more accurately and efficiently by offering real-time insights into individuals’ understanding of concepts (X. Liu, 2025). Studies also indicate that engaging students in conversations with GenAI about specific issues can broaden their thinking, encourage deeper, more critical questioning, and support a complete cognitive cycle (Nasr et al., 2025; K.-S. Tang et al., 2026). At the same time, GenAI’s impact depends significantly on students’ abilities to rigorously assess AI-generated content and use it appropriately. Without explicit instruction or strong AI literacy, students may misconstrue results, resort to surface-level learning, or develop an excessive reliance on automation, which undermines meaningful learning (Bewersdorff et al., 2025). Additionally, ethical concerns such as authorship, data privacy, and equity remain pressing, prompting crucial questions about the responsible adoption of GenAI in education (De Fine Licht, 2025).

Moreover, researchers use various theoretical models, such as the technology acceptance model, task-technology fit theory, theory of planned behavior, expectation confirmation theory, and unified theory of acceptance and use of technology, to explain how GenAI affects students’ learning behavior and underlying mechanisms (Z. Liu et al., 2025; Nikolic et al., 2024). Frequently discussed influencing factors include perceived usefulness, perceived ease of use, performance expectancy, facilitating conditions, perceived risk and trust, and effort expectancy (Diao et al., 2024; Granić, 2025).

These insightful studies have enriched our understanding of the relationship between GenAI and students’ learning behavior. However, there still exist two gaps in current studies. Firstly, most studies overlook students’ individual characteristics in the adoption of GenAI, focusing mainly on the technology’s influence. Most of the aforementioned research models emphasize objective factors introduced by technologies but neglect subjective factors such as emotions, motivations, and abilities. This limits a nuanced, context-sensitive analysis of GenAI’s impact on student learning behavior. Second, current research often uses linear methods, such as regression analysis and partial least squares, to investigate the link between GenAI and students’ learning behavior. This symmetric approach assumes uniform effects of external variables on the outcome variable and highlights similarities among antecedents (Purwanto & Sudargini, 2021.), but it disregards differences in students’ preferences and behavior.

To address the two gaps, the current study first adopts the COM-B model as the theoretical framework. The COM-B model is widely used in behavioral change research. It assumes that three fundamental components—capability, opportunity, and motivation—are essential for behavioral change and maintenance. This model provides a comprehensive understanding of student interactions with GenAI. It does this by capturing the dynamic interplay among students’ personal skills, environmental factors, and motivations. In addition, this study uses a mixed-methods approach. It combines CB-SEM and fsQCA to examine the sufficient and necessary conditions for those influencing factors. Using both linear and non-linear approaches enables the identification of distinct configuration paths that affect students’ learning behavior.

Therefore, this study aims to investigate the role of GenAI in transforming university students’ learning behavior and to examine the influencing factors within the COM-B model using a mixed-methods approach integrating CB-SEM and fsQCA. To achieve the research purpose, it focuses on two specific questions:

RQ1: How well does the COM-B model explain university students’ learning behavior to use GenAI?

RQ2: Which configurational paths of these factors can influence students’ learning behavior in using GenAI?

## 2. Literature Review and Hypotheses Development

### 2.1. Generative AI

Generative AI is a type of AI technology that creates new content based on the data it has learned (Dua & Patel, 2024). Since GenAI uses the information it acquires to generate original content, it can do more than just analyze data and make predictions. It also produces entirely new content (Katsamakas & Sanchez-Cartas, 2024). Previous studies have identified four key characteristics of GenAI. The first feature is creativity. GenAI can produce fresh, original content (Vinchon et al., 2024). This creativity relies on GenAI’s ability to study thousands of examples. It then combines them in new ways. The second characteristic is adaptability. After training, the GenAI system can perform various tasks and recognize general patterns in data. This ability enables it to transfer knowledge across different areas with minimal adjustment (Şahin & Karayel, 2024). Automation is another core feature. GenAI can potentially replace humans in tasks that involve manual labor (Alla, 2026). Automation offers many capabilities. It is like having a tireless employee who never rests. The last feature is personalization, which refers to GenAI’s ability to create content for humans based on their preferences or behaviors (Kakaraparthi, 2025).

The rapid advancement of GenAI is reshaping the education sector, transforming how students learn and access information. Many scholars view GenAI as a significant opportunity to redefine education, especially at the tertiary level. For instance, Andrade-Girón et al. systematically reviewed GenAI tools and their effects on student learning, concluding that these tools improve educational efficiency and engagement (Andrade-Girón et al., 2024). Berg et al. evaluated ChatGPT’s ability to aid educators in designing lessons and fostering critical thinking, finding that GenAI tools can facilitate pedagogical innovation (Van Den Berg & Du Plessis, 2023). Smolansky et al. explored educators’ and students’ views on GenAI’s impact on assessment, revealing varied attitudes and highlighting the need for assessment systems to evolve as AI’s role grows (Smolansky et al., 2023).

What’s more, current research indicates that various factors can affect the application of GenAI in the education sector. For instance, Nikolic et al. examined students’ attitudes toward GenAI in pedagogical and research contexts, utilizing multiple theoretical frameworks to comprehend learning behavior. Their results suggest that both immediate perceptions and long-term considerations influence students’ willingness to adopt GenAI, while also highlighting the importance of ethical and behavioral considerations in its implementation (Nikolic et al., 2024). What’s more, Al-Emran et al. investigated AI characteristics, such as perceived anthropomorphism, intelligence, and animacy, alongside behavioral theories to model social sustainability in the use of GenAI among university students. Their findings revealed that perceptions of GenAI’s social attributes significantly influence students’ learning behavior, underscoring the importance of GenAI’s human-like features in fostering learning engagement (Al-Emran et al., 2024). In addition, pedagogical beliefs and motivation also play vital roles. Batani et al. reported that performance expectancy, effort expectancy, social influence, facilitating conditions, and hedonic motivation all positively affect students’ intentions to utilize GenAI within educational settings (Silhavy & Silhavy, 2026). These factors suggest that students are more inclined to engage with GenAI when they perceive clear benefits, ease of use, social support, and enjoyment. Furthermore, Tang et al. identified key attitudinal dimensions, including societal concern, clarity of policy, fairness, trust, and potential career impacts, that influence the acceptance of GenAI tools (X. Tang et al., 2025). Together, these attitudes may either promote or impede the responsible integration of AI into learning behavior.

### 2.2. The COM-B Model

The COM-B model posits that, for an individual to undertake a specific behavior (B), they must possess the requisite capability (C), opportunity (O), and motivation (M) (Willmott et al., 2021). Modifying behavior requires altering at least one of these elements. Capability includes the mental and physical capacity to execute the behavior. Psychological capability covers necessary knowledge and mental skills, such as attention, memory, and decision-making. Physical capability refers to bodily functions like strength, stamina, or dexterity, required for a behavior. Opportunity includes external factors, both physical and social, that facilitate a behavior. Physical opportunity concerns the environment and resources, such as financial means or time. Social opportunity involves the actions of significant others and broader social networks. Motivation includes internal mental and emotional processes that promote or inhibit behavior and influence which action a person chooses. These processes may be reflective, involving plans, beliefs, attitudes, or goals. They may also be automatic, shaped by emotions and habits outside conscious awareness. The components of the COM-B model interact through positive and negative feedback loops, forming dynamic systems that govern behavior.

In the education field, many researchers have used the COM-B model to investigate and explain behavioral change. For example, several have combined the COM-B model with the Theoretical Domains Framework (TDF) to investigate barriers and facilitators to university students’ participation in physical activities. These studies collectively highlight the importance of interventions focusing on environmental context and resources, social influences, and goals—such as environmental changes, education, and enablement strategies (Brown et al., 2024; Ndupu et al., 2023). Furthermore, the COM-B model has also been increasingly used to investigate the application of emerging technologies for educational purposes. For instance, Mark E. Patterson conducted a pilot survey using the COM-B model to explore attitudes toward AI adoption in Southern African higher education. Key facilitators included strong infrastructure, readiness to address ethical issues, and belief in AI’s benefits; the main barrier was students’ ethical concerns about AI decision-making (Patterson et al., 2025). Likewise, Li’s study, guided by the COM-B theory, explored Chinese tertiary students’ motivations to start online businesses on live-streaming platforms, identifying various influencing factors across environmental opportunity and personal capability (L. Li et al., 2023). Despite these developments, there is limited research using the COM-B framework to explore students’ learning behavior with GenAI.

Currently, most studies use technology-adoption models, such as the Technology Acceptance Model (TAM), the Unified Theory of Acceptance and Use of Technology (UTAUT), and the Task-Technology Fit (TTF) theory, to examine students’ learning behavior with GenAI. These models provide a solid foundation for understanding what drives students to use GenAI and what factors influence their initial adoption. However, they don’t provide much insight into how students build skills, adapt to new resources, or keep using GenAI in the long run. Additionally, they overlook key factors such as social influence, individual capabilities, and the mix of deliberate and automatic motivation (FakhrHosseini et al., 2024). In contrast, the COM-B model examines more than just initial adoption; it explores the ongoing behaviors involved in using GenAI, such as iterative prompting and self-directed learning. Furthermore, this model integrates internal factors, such as capability and motivation, with external opportunities within a single framework. Examining feedback loops clearly demonstrates how improving skills can enhance both motivation and access to opportunities over time. As a result, applying this model to GenAI learning behavior offers a framework that is both theoretically solid and grounded in capturing the complex factors that influence students’ engagement.

### 2.3. Research Model and Hypotheses

#### 2.3.1. Competence

In this study, competence refers to the psychological and physical skills students need to use GenAI. It includes independent learning and receptive abilities. Independent learning ability is about the cognitive skills that help students develop learning strategies and select appropriate resources. It also enables them to refine interactions with GenAI to achieve academic goals. This competence is especially crucial during times of rapid technological change. As Huang points out, GenAI technology is evolving quickly. This change makes traditional passive teaching methods less effective (Huang et al., 2025). Therefore, students need to seek information, explore new features, master advanced techniques through trial and error, and solve problems independently. These activities rely heavily on mental functions such as memory, attention, problem-solving, and reasoning. Ma et al. confirm that students with strong independent learning skills are better at planning their learning paths. They also identify quality resources and continuously refine their interaction strategies with GenAI (Ma et al., 2025).

Second, receptive ability includes both psychological traits and physical adaptability. Psychologically, it covers students’ openness to new technologies, willingness to try new tools, and cognitive flexibility in adopting new learning methods. Physically, it involves motor skills and coordination to quickly learn new software functions and navigate various interfaces. A longitudinal study by Polyportis found that students initially skeptical of AI shifted their learning behavior after seeing peers use it successfully (Polyportis, 2024), highlighting the psychological role in AI acceptance. Additionally, Watson found that when AI output is unsatisfactory, highly receptive individuals engage in cued optimization rather than give up, showing cognitive flexibility (Mittal et al., 2024).

Building on the above discussion, this study proposes the following hypotheses:

**H1.**  *Students’ independent learning ability positively influences their learning behaviors with GenAI.*

**H2.**  *Students’ receptive ability positively influences their learning behaviors with GenAI.*

#### 2.3.2. Opportunity

This study defines opportunity as external factors outside the student’s control that either facilitate or hinder the use of GenAI. It includes both the learning environment and the availability of AI-support equipment, which together shape the context for adopting GenAI.

First, the learning atmosphere encompasses the socio-academic setting, including norms, expectations, and cultural attitudes, that influence perceptions and the use of GenAI. As Wijnhoven points out, adopting new technologies is not just a personal decision but is rooted in social and institutional contexts (Wijnhoven, 2022). In this context, students are influenced by peers, instructors, and policy attitudes toward ethical and acceptable AI use. For example, Divya found that students in supportive environments where GenAI use is openly discussed, encouraged, and guided show higher levels of proficient and innovative application (Law, 2024). Similarly, Chambers reported that clear institutional guidelines, along with instructor support and peer learning groups, significantly reduce student anxiety and boost confidence in using GenAI appropriately for academic tasks (Chambers & Owen, 2024).

Second, AI support equipment includes the tangible technical resources and infrastructure needed for access and use of GenAI. This covers reliable hardware such as computers and tablets, stable and affordable internet, and access to advanced GenAI tools, which may be limited by economic or institutional support. The Global Education Monitoring Report identified the “digital divide” as a major obstacle, highlighting that unequal access to high-performance computing and premium AI software results in significant disparities in learning opportunities (GEM Report UNESCO, 2023). Liu et al.’s research showed that students with university-provided, high-quality GenAI platforms and technical support experienced less frustration. They were more likely to use AI for complex, long-term projects than those who relied on free versions or faced frequent technical issues (Z. Liu et al., 2025).

Based on the above discussion, this study proposes the following hypotheses:

**H3.**  *Learning atmosphere positively influences students’ learning behavior with GenAI.*

**H4.**  *AI support equipment positively influences students’ learning behavior with GenAI.*

#### 2.3.3. Motivation

Motivation refers to the psychological factors that inspire, guide, and sustain students’ use of GenAI in educational settings. Unlike competence and opportunity, which relate to personal ability and external circumstances, motivation acts as the main force that determines whether students engage with GenAI, how consistently they use it, and their overall approach. The current study highlights two primary types of motivation: intrinsic and extrinsic.

Intrinsic motivation comes from the natural interest or enjoyment gained from using GenAI. Students motivated in this way participate because they find the interaction engaging, the exploration satisfying, or the problem-solving process intellectually rewarding. This motivation is linked to deeper involvement and more creative use. For example, according to Ryan & Deci’s Self-Determination Theory, intrinsic motivation encourages greater persistence and understanding (Ryan & Deci, 2000). Similarly, Mishra found that curiosity-driven students tend to use GenAI for complex tasks, such as generating and examining multiple perspectives in research, rather than just completing assignments (Mishra & Henriksen, 2025). Furthermore, Mazari’s study demonstrated that students spend more time refining prompts and show increased metacognitive awareness when incorporating AI feedback into their learning (Mazari, 2025).

In contrast, extrinsic motivation means using GenAI to achieve external goals, like earning better grades, meeting course requirements, or gaining recognition from peers and instructors. This motivation can encourage initial participation. However, it may not improve the quality or depth of learning. For example, Pan noted that rubric-based rewards significantly increase the frequency with which students use GenAI. Yet, these rewards do not necessarily promote critical thinking or creativity (Pan, 2025). Zhang et al. also found that students motivated by external rewards use GenAI mainly for practical tasks, such as paraphrasing and summarizing, with less interest in exploring its intellectual potential (Tian & Xiang, 2026).

Based on this understanding, the study puts forward the following hypotheses:

**H5.**  *Students’ intrinsic motivation positively influences their learning behaviors with GenAI.*

**H6.**  *Students’ extrinsic motivation positively influences their learning behaviors with GenAI.*

#### 2.3.4. AI Literacy

Long and Magerko defined AI literacy as a comprehensive set of skills. These skills enable individuals to critically evaluate and understand AI technologies. They also allow people to communicate effectively with AI systems, collaborate on AI-related tasks, and confidently use AI tools. These abilities apply across contexts such as digital platforms, homes, and workplaces (Long & Magerko, 2020). AI literacy includes not only cognitive and operational skills but also the capacity to interact and collaborate with AI systems (Ng et al., 2021). On the one hand, AI literacy supports academic, personal, and professional study by enabling students to use information and digital technologies safely and critically. On the other hand, it involves assessing, understanding, and creating information in various formats from multiple sources using digital tools and communication platforms. Prior studies have indicated that high levels of AI literacy help students develop positive attitudes toward technology adoption. They also reduce anxiety caused by technological uncertainty and enable greater flexibility and creativity in course learning and classroom engagement (Ayed et al., 2025; Schiavo et al., 2024). Similarly, Xu’s study shows that in AI-enhanced learning environments, students with higher AI literacy can navigate complex AI tools, interpret algorithmic outputs, and personalize learning experiences (Xu, 2024). In contrast, some researchers have found that students with lower AI literacy levels find AI tools challenging to use. As a result, they fail to achieve the intended learning outcomes (Bećirović et al., 2025; H. Li et al., 2025).

Specifically, students with greater competence but limited AI literacy might struggle to craft meaningful prompts, assess AI outputs, or incorporate feedback into their learning. This can lead to superficial or inefficient behavior. In contrast, students with high AI literacy can better align their cognitive skills with AI functionalities. What’s more, although universities offer numerous AI resources, access alone doesn’t guarantee productive learning. Students with lower AI literacy may experience cognitive overload, uncertainty, or misuse of AI tools, leading to superficial engagement. Conversely, students with higher AI literacy may recognize AI’s capabilities and limitations. They can choose appropriate tools for tasks and use resources strategically. Additionally, motivated students may eagerly adopt AI tools, but insufficient literacy can lead to shortcut-taking or ethically questionable use. This reduces learning quality. High AI literacy helps motivated students maintain autonomy and mastery by using GenAI as a supportive partner. Thus, lacking adequate AI literacy may weaken or even negate the positive influence of competence, opportunity, and motivation on learning behaviors.

Therefore, AI literacy encompasses not just cognitive skills but also attitudes and behaviors that directly influence students’ engagement with GenAI tools. By shaping the connection between learning abilities and learning behavior, AI literacy is pivotal in ensuring GenAI is used responsibly and effectively. Further investigation into how AI literacy relates to other factors in the COM-B model can offer deeper insights into the conditions under which GenAI is most effective in improving student learning.

Based on the above discussion, this study predicts that:

**H7.**  *AI literacy can act as a moderating role between (a) students’ independent learning ability, (b) students’ receptive ability, (c) learning atmosphere, (d) AI support equipment, (e) students’ intrinsic motivation, (f) students’ extrinsic motivation, and students* *’ learning behavior.*

**Figure 1:**
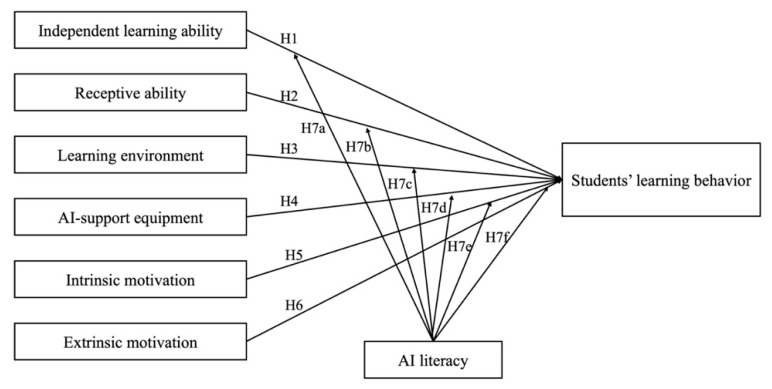
The research model and hypotheses.

Figure 1 shows the research model and hypotheses.

## 3. Methods

### 3.1. Measuring Instrument

This study used an online survey for the investigation. We developed a questionnaire (see Appendix A) adapted from previous studies to collect research data. Specifically, the first three items measuring independent learning ability were adapted from Qi’s research (Qi et al., 2025). The next three items, which assess students’ receptive ability, were cited from Keyworth’s research (Keyworth et al., 2020). We measured the learning environment, AI-support equipment, and students’ learning behavior using Drattell’s scale (Drattell et al., 2025). Students’ intrinsic and extrinsic motivation were measured with Daryabeygi-Khotbehsara’s scale (Daryabeygi-Khotbehsara et al., 2024). In addition, we assessed AI literacy with three items adapted from Börekci and Çelik’s study (Börekci & Çelik, 2024). Each item used a 5-point Likert scale, ranging from 1 (strongly disagree) to 5 (strongly agree). We invited five experts in higher education research to evaluate the questionnaire’s quality. They reviewed its readability, structure, reliability, and validity. The experts provided useful feedback and suggestions. We used these to revise the questionnaire. Afterward, we conducted a pilot test by inviting 54 university students proficient in GenAI to complete the questionnaire. Pilot test results showed high reliability (Cronbach’s α values above 0.80 for all constructs) and validity (AVE values exceeding 0.50 for all constructs).

### 3.2. Data Collection

We used an online platform to collect the data. Participants were students from five Chinese universities. Before the participants answered the questionnaire, they needed to answer a screening question: Have you ever used GenAI, such as ChatGPT, DeepSeek, Dola, and Kimi, etc., in your learning process? Those who responded “Yes” were invited to continue answering the questionnaire. All procedures followed in this study comply with the principles outlined in the Declaration of Helsinki. We obtained informed consent from all participants, provided a clear explanation of the research purpose, and emphasized the principles of data confidentiality. Data was collected from 17 October to 3 December 2025, with an initial 487 data points. We checked each response and excluded 24 that exhibited low variance. Consequently, 463 responses were considered reliable for further analysis. We also examined the dataset for univariate and multivariate outliers to ensure its suitability for the following statistical analysis. The tests revealed no univariate outliers, but 15 responses were identified as multivariate outliers. As a result, we excluded these 15 responses, leaving a final sample of 438. Table 1 shows the participants’ demographic information.

### 3.3. Research Methods

We employed CB-SEM and fsQCA methods to analyze the collected data. CB-SEM is a confirmatory statistical method that evaluates theoretical models by examining the observed data’s covariance matrix. CB-SEM aims to minimize the discrepancies between observed and model-implied covariance matrices. It primarily uses Maximum Likelihood (ML) estimation. It is ideal for theory testing, model comparison, and assessing model fit in confirmatory research (Dash & Paul, 2021). The current research model, comprising seven constructs, is intricate and is adapted from the COM-B framework. Therefore, CB-SEM is essential to assess the model’s symmetry and linearity. It also helps quantify the strength and direction of causal relationships and evaluate overall model quality. FsQCA, on the other hand, is an asymmetrical approach that analyzes complex causal patterns across diverse cases. FsQCA identifies logical connections among various causal factors and an outcome and then generates rules indicating which subsets of these factors are sufficient to produce the outcome (Nikou et al., 2024). FsQCA methodology captures equifinality and reveals nonlinear relationships. It uncovers multiple sufficient conditions that can lead to tourists’ high intention to adopt AI devices. Therefore, using both CB-SEM and fsQCA analyses facilitates the assessment of the complex research model and provides insights into the relational intricacies among the constructs.

## 4. Results

### 4.1. The Results of CB-SEM Analysis

#### 4.1.1. The Results of the Construct Measurement

We used SPSS 26.0 and AMOS 24.0 to conduct the CB-SEM analysis. First, we conducted a test for common method bias to address potential bias and enhance the robustness and validity of the findings. We used Harman’s single-factor test to check for common method bias. The result indicated that the first principal component had an eigenvalue greater than 1 and explained 26.13% of the variance. This was well below the 40% threshold, suggesting that common method bias was not a significant issue in this study.

Subsequently, we conducted confirmatory factor analysis (CFA) to assess the reliability and validity of the constructs. Table 2 presents the CFA results. As illustrated, all the constructs’ factor loadings exceed 0.8, surpassing the recommended minimum threshold of 0.7. Furthermore, the findings for composite reliability (CR) and average variance extracted (AVE) indicate that all constructs have satisfactory values, with CR exceeding 0.7 and AVE exceeding 0.5. Additionally, the study evaluated the constructs’ convergent and discriminant validity. For convergent validity, an AVE value greater than 0.5 is required; all constructs met this criterion, indicating excellent convergent validity. To establish discriminant validity, it is essential that the diagonal entries of each latent variable exceed the highest correlation associated with that variable. Table 3 shows the bolded square roots of the AVEs for each construct on the diagonal, all of which exceed the corresponding correlation coefficients, confirming that the discriminant validity conditions are satisfied.

#### 4.1.2. The Results of Hypothesis Tests

The results of the data analysis indicate that the structural model has a good fit (CMIN = 221.53, DF = 83, CMIN/DF = 3.05, *p* = 0.00, GFI = 0.94, NFI = 0.91, RFI = 0.90, IFI = 0.96, TLI = 0.91, CFI = 0.92, RMSEA = 0.078). Table 4 illustrates the model’s path analysis results. Specifically, hypotheses 1(β = 0.21, *p* < 0.001), 2(β = 0.24, *p* < 0.001), 4(β = 0.22, *p* < 0.001), 5(β = 0.19, *p* < 0.001), 6(β = 0.28, *p* < 0.001) has significant influence on students’ learning behavior. However, hypothesis 3(β = 0.17, *p* = 0.429) shows no positive impact on students’ learning behavior.

We further examined the moderating role of AI literacy between GenAI and students’ learning behavior. The moderation analysis involves testing the path model with independent variables, the moderator, and the interaction variable. The results of the moderating effect test demonstrate that AI literacy can moderate the relationship between GenAI and students’ learning behavior (Table 5). Therefore, Hypotheses 7a–7f are supported.

### 4.2. The Results of fsQCA

In the initial phase of the fsQCA analysis, it is imperative to calibrate the outcome variable and all causal conditions. Each variable is directly calibrated, necessitating the specification of three anchors: full membership, the crossover point, and nonmembership. In this study, calibration is conducted using the percentiles (90, 50, and 10) from the 5-point Likert scale in the research questionnaire. Additionally, the membership score of exactly 0.5 presents analytical challenges, which aligns with Fiss’s recommendation to add 0.001 to all variables with a membership score below one.

The next step is to address the necessary conditions. Based on Ragin, a factor is considered necessary if its consistency is 0.9 or higher (Ragin, 2006). As seen in Table 6, no condition meets this threshold, indicating that there are no necessary conditions.

Then, we analyzed the sufficient conditions. The analysis of sufficient conditions initiates with the construction of the truth table. The truth table employed in this study comprises 2^k rows, where k denotes the number of conditions. Each row represents a distinct configuration of these conditions. The frequency and consistency thresholds are applied to evaluate the truth table. Consistent with the methodology of Krogslund et al., the frequency threshold is set at 1, and the consistency threshold at 0.8 (Krogslund et al., 2015). Additionally, the Proportional Reduction in Inconsistency (PRI) threshold is established at 0.75. When the truth table is analyzed using fsQCA 4.1 software, three types of solutions are generated: complicated, moderate, and parsimonious. This research employs intermediate solutions, which are a subset of complicated solutions and also incorporate parsimonious solutions.

Table 7 presents the results of the intermediate fsQCA solution. Specifically, there are seven configurations that contribute to students’ learning behavior. The solution coverage rate is 0.882, meaning these seven pathways explain most students’ learning behavior. The overall consistency score is 0.864. Consistency scores above 0.74 indicate insightful solutions from the integrated evaluation. Consequently, this study offers a robust model, as all configurations have a consistency score exceeding 0.90. Among these, configurations 2, 3, and 5 are most crucial, with consistency scores above 0.95. Configuration 3 showed the highest consistency (0.975) and explained many cases (raw coverage: 0.529), making it the best solution for improving learning behavior. It indicated that receptive ability (core condition), independent learning ability, AI support, intrinsic motivation, and the absence of learning atmosphere and extrinsic motivation influence students’ learning behavior. Configuration 2 had a high consistency (0.968) and significant coverage (0.413), suggesting that the outcome could be achieved with AI literacy (core condition), receptive ability, intrinsic motivation, and no AI support. Lastly, configuration 5 demonstrated that receptive ability (core condition), AI literacy (core condition), independent learning ability, learning atmosphere, and intrinsic motivation lead to high consistency (0.966) and good coverage (0.362).

## 5. Discussion

The results of the CB-SEM analysis show that students’ independent learning ability, receptive ability, AI support equipment, intrinsic motivation, and extrinsic motivation can significantly impact students’ learning behavior. These results correspond with previous research findings. For instance, Qi’s research found that university students’ capabilities, including critical thinking, self-directed learning ability, and AI literacy, can positively influence the quality of information obtained from GenAI tools (Qi et al., 2025). George’s findings emphasized that AI could enhance students’ autonomy and stimulate creativity by upgrading and enriching the learning environment (George, 2023). Furthermore, Zhang’s research examined how intrinsic motivation and TAM-related factors influence undergraduates’ acceptance of ChatGPT for active learning (Zhang et al., 2024). The results revealed that students’ intrinsic motivation, perceived usefulness, and ease of use can positively influence their learning behavior. However, the learning atmosphere did not exert a positive influence on students’ learning behavior. This result contradicts Hu’s research, which indicates that an AI-supported smart learning environment can substantially enhance students’ learning effectiveness and outcomes (Y.-H. Hu, 2022). A possible explanation is that when AI-enhanced environments are not effectively integrated with pedagogical methods and learning tasks, their impact on actual learning behavior remains limited. It is also possible that students’ personal factors outweigh environmental influences, making individual motivation more pivotal than the learning environment itself. The findings also demonstrated that AI literacy can moderate the relationship between GenAI and students’ learning behavior. This result aligns with Hidayat-ur-Rehman’s study, which suggests that AI literacy not only moderates the relationship between AI tool use and learning benefits but also enhances students’ motivation and engagement in AI-mediated learning environments (Hidayat-ur-Rehman, 2024). Tiandem-Adamou’s study also confirmed that students’ co-constructive interactions with GenAI depend on their understanding of AI literacy. This highlights the moderating role of AI literacy in fostering meaningful human–AI collaboration (Tiandem-Adamou, 2025).

The fsQCA analysis shows that seven configurations significantly influence students’ learning behavior. The results indicate that a single solution is not the best approach for positively shaping students’ learning behavior. These seven configurations can be categorized into three groups. The first group includes the three-core condition paths. For example, configuration 1 involves independent learning ability, AI support equipment, and intrinsic motivation. The second group consists of single-core condition paths. Configurations 2 and 3 each have one core condition. The third group has double-core condition paths. Configurations 4, 5, 6, and 7 each involve two core conditions. The findings align with a previous study by Li and Wang. Their work also highlights that students’ use of GenAI depends on multiple factors, with no single factor being decisive (Wang et al., 2025). This configurational approach emphasizes the concept of equifinality in GenAI-supported learning. It suggests that students can adopt various pathways to effective learning rather than relying on a single mechanism. Differences between symmetric and asymmetric analyses reveal the complexity of students’ learning behavior. These differences illustrate the limitations of relying solely on symmetric approaches for a comprehensive understanding. Therefore, the results underscore the importance of asymmetrical analysis. They also caution against relying only on symmetrical methods when planning future strategies. Overall, the fsQCA findings reinforce the conclusions from the CB-SEM analysis.

## 6. Conclusions

### 6.1. Theoretical Implications

Firstly, numerous studies have examined the impact of GenAI on students’ learning behavior. However, few have used the COM-B model as a theoretical framework. This model offers a comprehensive understanding of learning behavior by including both individual qualities and environmental factors. The results show that internal and external factors can significantly shape students’ learning behavior with GenAI. As a result, the current research extends insights from studies on students’ learning behavior with GenAI and deepens understanding of the mechanisms through which GenAI affects human behavior.

Secondly, this study employed a mixed-method approach to investigate the relationship between GenAI and students’ learning behavior. The findings from fsQCA suggest that various configurations can positively impact students’ learning behavior with GenAI. Few prior studies have addressed the complex interactions among antecedents and their combined effects on student behavior. The seven configurations illustrate that students’ learning behavior with GenAI is intricate and that different factors can be combined to influence it. The integration of diverse factors introduces a theoretical perspective and innovative methodology to the existing literature and provides a foundational basis for researchers exploring students’ learning behavior with GenAI.

### 6.2. Practical Implications

First, the results of this study offer specific managerial implications for universities. The findings provide empirical support for universities and educators to proactively adopt GenAI, as it meaningfully shapes students’ learning behavior. Universities should upgrade their digital teaching infrastructure and offer comprehensive technical support, both hardware and software, to help students use GenAI. The factors identified in this study may serve as actionable guidelines for universities to implement practical strategies to facilitate and sustain GenAI integration for educational advancement. For example, universities can design targeted training programs or workshops to enhance students’ AI literacy, enabling them to use GenAI tools more skillfully and critically in academic settings. Additionally, the COM-B model provides a conceptual framework for teachers to interpret students’ learning behavior in the context of GenAI. Thus, teachers can leverage these positive factors to maximize students’ educational outcomes through GenAI.

Second, this study shows that AI literacy can influence the relationship between GenAI and students’ learning behavior within the COM-B framework. Therefore, it is necessary to enhance students’ AI literacy in daily studies. For example, universities can create curricula to introduce the basic principles, capabilities, and limitations of GenAI tools. These courses can help by improving technical understanding and critical evaluation skills for GenAI systems. In addition, universities can offer hands-on practices with GenAI to help students build practical competence and sound usage strategies. When developing students’ AI literacy, universities should address students’ competence, external opportunities, and individual motivations. This is important because learning behavior emerges from the interaction of internal psychological factors and external environmental conditions, as described in the COM-B model.

Thirdly, the results of the fsQCA analysis identify seven configurations that can enhance students’ learning behavior with GenAI. This finding offers actionable guidance for implementing GenAI in universities, which vary in adoption levels. Some have already embedded GenAI tools into teaching, learning support, and administration, while others are still exploring initial implementation. Therefore, instead of adopting a uniform approach, universities can use these configurations to strategically deploy or refine GenAI, tailored to their institutional needs and student profiles. For instance, universities at an early adoption stage can focus on configurations that stress foundational elements, such as ensuring access to GenAI and setting clear guidelines to build a supportive learning environment. Conversely, universities with advanced GenAI integration can employ configurations that combine strong student capability, ample learning opportunities, and robust motivational factors. This configurational approach allows universities to flexibly choose and tailor GenAI strategies, optimizing educational outcomes.

### 6.3. Research Limitations and Future Research Suggestions

While this study offers valuable theoretical and practical insights into factors influencing students’ learning behavior with GenAI, several limitations should be addressed in future research. First, since all participants were from Chinese universities in highly developed areas of the eastern region and shared similar educational and cultural backgrounds, the findings may be biased. This highlights a gap, as GenAI adoption can vary significantly between developed and underdeveloped regions. To address this, future studies should include more diverse samples, especially from underdeveloped regions and varied cultural backgrounds. Second, although the CB-SEM analysis showed that the learning atmosphere did not positively influence students’ learning behavior, the study did not delve into detailed reasons for this. As a result, the authors of future research could carry out qualitative studies to investigate the underlying causes.

## Figures and Tables

**Table 1:** The profile of the participants.

Variables	N (%)	Variables	N (%)
Gender		Frequency of GenAI Use	
Female	226 (51.6)	Everyday	194 (44.3)
Male	212 (48.4)	3–4 times per week	223 (51)
Grade Level		1–2 times per week	21 (4.7)
Freshman	87 (19.7)	Purpose of Using GenAI	
Sophomore	136 (31.2)	Coursework assistance	357 (81.5)
Junior	109 (24.9)	Information search	204 (46.6)
Senior	106 (24.2)	Writing support	146 (33.3)
Major		Brainstorming	132 (30.1)
Humanities	143 (32.5)	Language translation	68 (16)
Sciences	124 (28.3)	Creative content creation	84 (19.1)
Engineering	112 (25.7)	Data analysis	182 (41.6)
Arts	59 (13.5)	Programming	108 (24.7)
Region of Residence			
Eastern China	165 (37.7)		
Western China	73 (16.7)		
Central China	84 (19.1)		
Southern China	116 (26.5)		

**Table 2:** The results of the reliability and convergent validity.

Construct	SD	FL	S.E.	z-Value	Cronbach α	AVE	CR
Independent learning ability					0.886	0.626	0.887
ILA1	1.115	0.882	0.063	13.187			
ILA2	1.123	0.864	0.062	12.139			
ILA3	1.168	0.893	0.071	13.470			
Receptive ability					0.852	0.703	0.864
RA1	1.182	0.871	0.054	18.563			
RA2	1.147	0.879	0.061	15.863			
RA3	1.113	0.888	0.059	16.241			
Learning atmosphere					0.874	0.661	0.891
LA1	1.201	0.891	0.064	18.010			
LA2	1.174	0.885	0.075	17.826			
LA3	1.155	0.873	0.069	16.533			
AI support equipment					0.882	0.694	0.872
AI-SE1	1.117	0.896	0.058	17.172			
AI-SE2	1.149	0.841	0.059	18.935			
AI-SE3	1.140	0.872	0.064	17.532			
Intrinsic motivation					0.871	0.727	0.881
IM1	1.192	0.890	0.053	11.435			
IM2	1.165	0.895	0.053	14.660			
IM3	1.183	0.887	0.062	17.033			
Extrinsic motivation					0.876	0.658	0.883
EM1	1.161	0.855	0.072	13.645			
EM2	1.152	0.862	0.053	18.480			
EM3	1.173	0.849	0.074	17.769			
AI literacy					0.826	0.744	0.904
AI-L1	1.139	0.877	0.055	10.153			
AI-L2	1.122	0.893	0.070	9.659			
AI-L3	1.110	0.881	0.071	11.309			
Learning behavior					0.869	0.701	0.882
LB1	1.204	0.899	0.052	18.504			
LB2	1.273	0.887	0.051	18.861			
LB3	1.242	0.886	0.057	16.036			

**Table 3:** The result of discriminant validity.

Construct	ILA	RA	LA	AI-SE	IM	EM	AI-L	LB
ILA	**0.791**							
RA	0.582	**0.838**						
LA	0.536	0.714	**0.813**					
AI-SE	0.577	0.692	0.582	**0.833**				
IM	0.614	0.588	0.495	0.602	**0.853**			
EM	0.502	0.526	0.514	0.499	0.573	**0.811**		
AI-L	0.583	0.551	0.583	0.552	0.580	0.662	**0.863**	
LB	0.561	0.568	0.529	0.571	0.564	0.531	0.539	**0.837**

Note: The bold numbers on the diagonal represent the root of AVE.

**Table 4:** The structural model path analysis.

Hypothesis	Path	Estimate	S.E.	C.R.	*p*-Value	Result
H1	ILA → LB	0.21	0.060	3.500	***	Supported
H2	RA → LB	0.24	0.058	4.000	***	Supported
H3	LA → LB	0.17	0.213	0.789	0.429	Unsupported
H4	AI-SE → LB	0.22	0.058	3.793	***	Supported
H5	IM → LB	0.19	0.059	3.220	***	Supported
H6	EM → LB	0.28	0.093	3.011	***	Supported

Note: *** *p* < 0.001.

**Table 5:** The results of the moderating effect test.

Hypothesis/Path	β Coefficient	t-Value	Moderation
H7a: ILA × AI literacy → LB	0.08	4.26 ***	Yes
H7b: RA× AI literacy → LB	0.04	4.74 ***	Yes
H7c: LA × AI literacy → LB	0.02	2.19 ***	Yes
H7d: AI-SE × AI literacy → LB	0.07	5.03 ***	Yes
H7e: IM × AI literacy → LB	0.03	4.46 ***	Yes
H7f: EM × AI literacy → LB	0.07	5.27 ***	Yes

Note: *** *p* < 0.001.

**Table 6:** Analysis of necessary conditions.

Constructs	Consistency	Coverage
Independent learning ability	0.735	0.727
~ Independent learning ability	0.792	0.761
Receptive ability	0.782	0.751
~ Receptive ability	0.793	0.694
Learning atmosphere	0.817	0.705
~ Learning atmosphere	0.774	0.691
AI support equipment	0.822	0.784
~ AI support equipment	0.713	0.761
Intrinsic motivation	0.789	0.683
~ Intrinsic motivation	0.802	0.711
Extrinsic motivation	0.799	0.696
~ Extrinsic motivation	0.837	0.742
AI literacy	0.775	0.736
~ AI literacy	0.803	0.793

**Table 7:** Configurations for students’ learning behavior.

Configuration	C1	C2	C3	C4	C5	C6	C7
Independent learning ability	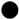		●	⊗	●	●	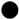
Receptive ability	●	●	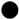	⊗	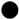	⊗	●
Learning atmosphere			⊗	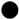	●	⊗	⊗
AI support equipment	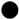	⊗	●				⊗
Intrinsic motivation	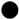	●	●	●	●	●	●
Extrinsic motivation			⊗			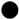	●
AI literacy	⊗	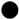		●	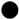	⊗	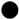
**Consistency**	0.931	0.968	0.975	0.936	0.955	0.942	0.917
**Raw coverage**	0.462	0.413	0.529	0.303	0.436	0.506	0.338
**Unique coverage**	0.116	0.003	0.007	0.006	0.019	0.021	0.008
**Solution consistency**	0.864						
**Solution coverage**	0.882						

Note: 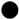 means the existence of core conditions; ● means the existence of edge conditions; ⊗ means the absence of edge conditions; the space means that the condition can either appear or be absent.

## Data Availability

Data are not publicly available due to proprietary restrictions and their planned use in ongoing subsequent research but are available from the corresponding author upon reasonable request.

## Appendix A. Questionnaire Items

Independent Learning Ability (ILA)

ILA1: I actively seek ways to use GenAI to solve learning problems on my own.

ILA2: I use GenAI to find valuable learning resources based on my learning needs.

ILA3: I can effectively manage my time when using GenAI for academic tasks.

Receptive Ability (RA)

RA1: I am willing to adjust my learning strategies based on suggestions provided by GenAI.

RA2: I feel comfortable integrating GenAI into my regular study activities.

RA3: I am open to trying new ways of learning with GenAI tools.

Learning Atmosphere (LA)

LA1: A supportive learning atmosphere encourages me to use GenAI tools in my studies.

LA2: My instructors’ positive attitudes toward GenAI motivate me to engage with it in coursework.

LA3: When my classmates actively use GenAI, I am more likely to use it for learning.

AI Support Equipment (AI-SE)

AI-SE1: The availability of AI-supported equipment encourages me to use GenAI for learning.

AI-SE2: Reliable access to AI-enabled equipment increases my frequency of using GenAI in academic tasks.

AI-SE3: The quality of AI-related hardware and software enhances my willingness to integrate GenAI into my studies.

Intrinsic motivation (IM)

IM1: I incorporate GenAI into my study activities because I find it personally meaningful.

IM2: I use GenAI out of curiosity about its capabilities.

IM3: I feel satisfied when I discover new insights through GenAI.

Extrinsic Motivation (EM)

EM1: I use GenAI in my studies because it helps me achieve better grades.

EM2: I am encouraged to engage with GenAI when its use is linked to course assessment or academic outcomes.

EM3: I am more likely to use GenAI when it improves my efficiency in completing assignments.

AI literacy (AI-L)

AI-L1: I can effectively design prompts to obtain useful results from GenAI tools.

AI-L2: I know the limitations and potential risks of using GenAI in academic work.

AI-L3: I understand how GenAI systems generate responses and content.

Learning Behavior (LB)

LB1: I will continue to use GenAI tools to support my academic learning.

LB2: I plan to increase my use of GenAI tools in the future.

LB3: I am willing to learn how to use GenAI tools more effectively.
